# *Pichia pastoris* regulates its gene-specific response to different carbon sources at the transcriptional, rather than the translational, level

**DOI:** 10.1186/s12864-015-1393-8

**Published:** 2015-03-11

**Authors:** Roland Prielhofer, Stephanie P Cartwright, Alexandra B Graf, Minoska Valli, Roslyn M Bill, Diethard Mattanovich, Brigitte Gasser

**Affiliations:** Department of Biotechnology, BOKU University of Natural Resources and Life Sciences Vienna, Muthgasse 18, 1190 Vienna, Austria; Austrian Centre of Industrial Biotechnology (ACIB), Muthgasse 11, 1190 Vienna, Austria; School of Life and Health Sciences, Aston University, Aston Triangle, Birmingham, B4 7ET UK; School of Bioengineering, University of Applied Sciences FH Campus Wien, Vienna, Austria

**Keywords:** *Pichia pastoris*, Methylotrophic yeast, Crabtree-negative yeast, Polysome profiling, Microarray analysis, Transcriptome, Glucose repression, Carbon substrate repression, Methanol induction

## Abstract

**Background:**

The methylotrophic, Crabtree-negative yeast *Pichia pastoris* is widely used as a heterologous protein production host. Strong inducible promoters derived from methanol utilization genes or constitutive glycolytic promoters are typically used to drive gene expression. Notably, genes involved in methanol utilization are not only repressed by the presence of glucose, but also by glycerol. This unusual regulatory behavior prompted us to study the regulation of carbon substrate utilization in different bioprocess conditions on a genome wide scale.

**Results:**

We performed microarray analysis on the total mRNA population as well as mRNA that had been fractionated according to ribosome occupancy. Translationally quiescent mRNAs were defined as being associated with single ribosomes (monosomes) and highly-translated mRNAs with multiple ribosomes (polysomes). We found that despite their lower growth rates, global translation was most active in methanol-grown *P. pastoris* cells, followed by excess glycerol- or glucose-grown cells. Transcript-specific translational responses were found to be minimal, while extensive transcriptional regulation was observed for cells grown on different carbon sources. Due to their respiratory metabolism, cells grown in excess glucose or glycerol had very similar expression profiles. Genes subject to glucose repression were mainly involved in the metabolism of alternative carbon sources including the control of glycerol uptake and metabolism. Peroxisomal and methanol utilization genes were confirmed to be subject to carbon substrate repression in excess glucose or glycerol, but were found to be strongly de-repressed in limiting glucose-conditions (as are often applied in fed batch cultivations) in addition to induction by methanol.

**Conclusions:**

*P. pastoris* cells grown in excess glycerol or glucose have similar transcript profiles in contrast to *S. cerevisiae* cells, in which the transcriptional response to these carbon sources is very different. The main response to different growth conditions in *P. pastoris* is transcriptional; translational regulation was not transcript-specific. The high proportion of mRNAs associated with polysomes in methanol-grown cells is a major finding of this study; it reveals that high productivity during methanol induction is directly linked to the growth condition and not only to promoter strength.

**Electronic supplementary material:**

The online version of this article (doi:10.1186/s12864-015-1393-8) contains supplementary material, which is available to authorized users.

## Background

*Pichia pastoris* (syn. *Komagataella* sp.) is a methylotrophic yeast that is widely used for the production of heterologous proteins and metabolites; it is also used as a model organism for the study of peroxisome biosynthesis and degradation, as well as for the analysis of protein secretion (see [1], and references therein). Its ability to use methanol as a carbon and energy source, its non-fermentative utilization of glucose and its efficient growth on glycerol are key metabolic features that make it attractive for bioprocess development.

Recently, Liang et al. [2] comprehensively annotated the *P. pastoris* transcriptome and identified novel untranslated regions (UTR), alternative splicing sites (AS), internal ribosome entry sites (IRES), upstream ATGs (uATGs) and upstream ORFs (uORFs). Transcriptional profiling of a recombinant strain harboring *Rhizomucor miehei* lipase (RML) under the control of the methanol-driven P_AOX1_ promoter revealed that cells grown on methanol induce genes involved in protein production and energy metabolism more than cells grown on glycerol. Methanol utilization takes place in peroxisomes; genes such as the alcohol oxidases (*AOX1, AOX2*), formaldehyde dehydrogenase (*FLD*), dihydroxyacetone synthase (*DAS1, DAS2*) and peroxisomal genes (e. g. *PEX1*) were all found to be induced on methanol.

The specific growth rate of a culture, which was kept constant in the study by Liang et al. [2], is also known to play a fundamental role in gene regulation and consequently in protein production. High growth rates were previously suggested to be beneficial for protein production in *P. pastoris* due to the up-regulation of genes related to gene expression and translation, while catabolic processes (e.g. autophagy, transport to the peroxisome and mitochondrial degradation, many of them under the control of *TOR* signalling), were shown to correlate negatively with increasing growth rate [3].

Less is known about the specific regulation of carbon substrate utilization, with the notable exception of *Saccharomyces cerevisiae*. Most studies in *S. cerevisiae* have been performed on glucose-grown cells under respiro-fermentative or fermentative growth conditions [4] or on non-fermentable carbon-sources such as glycerol or galactose. The shift from glucose to glycerol leads to extensive transcriptomic remodelling [5], a global translational down-regulation [6] and reduced growth rates. In contrast, the Crabtree-negative yeast, *P. pastoris*, maintains its respiratory metabolism even under conditions of excess glucose (such as that used in batch cultivations) and exhibits similar growth rates and substrate uptake kinetics when grown on either glucose or glycerol [7]. Shifts from glycerol to methanol, which is metabolized even more slowly with lower maximal specific growth rates, are often used in bioprocesses that employ *P. pastoris*.

Transcriptional regulators involved in glucose repression have been identified and studied in the methylotrophic yeasts *P. pastoris* and *Hansenula polymorpha*, and in the lactose-utilizing yeast *Kluyveromyces lactis* [8-13]. Glucose repression of methanol utilization genes is established as a feature of methylotrophic yeasts such as *Candida boidinii*, *H. polymorpha*, *Pichia methanolica*, and *P. pastoris* [14], but the degree of repression/de-repression by different carbon sources is species-dependent. For example, different modes of regulation have been described for key enzymes of methanol metabolism pathways such as alcohol oxidase, dihydroxyacetone synthase and formaldehyde dehydrogenase (summarized in [14,15]). Understanding the molecular mechanisms underpinning the unique carbon substrate utilization properties of *P. pastoris* is now required in order to more fully understand this valuable host organism.

The regulation of gene expression is often analyzed at the level of transcription, although it is well established that altered transcript levels are not necessarily reflected by the corresponding protein levels [16]. For example, the protein level of more than 70% of *S. cerevisiae* protein-coding genes is transcriptionally regulated, but this drops to only about 50% in *E. coli* [17] and is even lower in humans [18]. In order to obtain a more complete view of the regulation of gene expression in *P. pastoris*, we analyzed both transcriptional and translational responses of cells grown in glucose-, glycerol- or methanol-containing media. Microarray analysis was done on the total mRNA pool as well as on mRNAs that had been fractionated based upon ribosome occupancy. We adapted published methods for polysome profiling [6,19]: translationally quiescent mRNAs were defined as being associated with single ribosomes (monosomes); actively-translated mRNAs with multiple ribosomes (polysomes) [20]. The hybridization of a microarray with these mRNA fractions as well as the total mRNA population provided insight into how efficiently individual mRNA translation and global transcriptional responses are affected by carbon source utilization.

## Results and discussion

*P. pastoris* strain X-33 was cultivated in shake flasks under four different bioprocess conditions (Table 1): excess glycerol or glucose (batch culture conditions; these cells were harvested during exponential growth); limiting glucose (using slow glucose-releasing silica disks or feed beads in fed-batch mode, [21,22]); and periodic methanol addition (methanol induction conditions). Cells grown in excess glucose or glycerol or those grown in methanol had growth rates close to μ_max_: 0.23 h^−1^ for the former and 0.1 h^−1^ for the latter conditions. Cells in limiting glucose conditions grew at μ = 0.015 h^−1^.

**Table 1:** ***Pichia pastoris*** **cultivations in buffered synthetic media supplemented with different carbon substrates**

**Condition**	**ID**	**Start-OD**_**600**_	**Cultivation substrate**	**Cultivation time [h]**	**Harvest-OD**_**600**_	**μ [h** ^**−1**^ **]**	**Bioprocess Step**	**Replicates**
**Excess glucose**	D	0.1	2% glucose	23.3	10.0 (1.0)	0.23 (0.004)	Glucose batch	3
**Excess glycerol**	G	0.1	2% glycerol	23.3	10.5 (1.3)	0.23 (0.001)	Glycerol batch	3
**Methanol feed**	M	1.5	0.5 and 0.6% methanol	24.5	8.6 (1.4)	0.10 (0.008)	Methanol shot/feed	3
**Limiting glucose**	X	1.5	0.25% glucose and feed beads	16.8	11.4 (0.6)	0.010 - 0.022	Glucose fed batch	3

Cultures with different biomass densities were fed with appropriate amounts of carbon substrate in order that the cells could be harvested at a similar OD_600_ [mean (sd)]. Growth rates (μ) [mean (sd)] were recorded; the values were highly reproducible and reflect growth of typical bioprocess phases, as shown.

For polysome fractionation, cells were treated with cycloheximide, harvested and quickly chilled for sample preparation. Isolates were used for polysome profiling to obtain the profile data and to collect mono- and polysome fraction samples for mRNA extraction. mRNA was isolated from the fractionated and unfractionated isolates for microarray analysis; for each condition three biological replicates were analyzed.

The excess glucose condition, which is often used as a control for studies in *S. cerevisae*, was used as a control in our experiments.

### Global transcript profiles are very similar for excess glucose or glycerol grown *P. pastoris* cells, while extensive transcriptional regulation is observed for cells grown on methanol or limiting glucose concentrations

**Figure 1:**
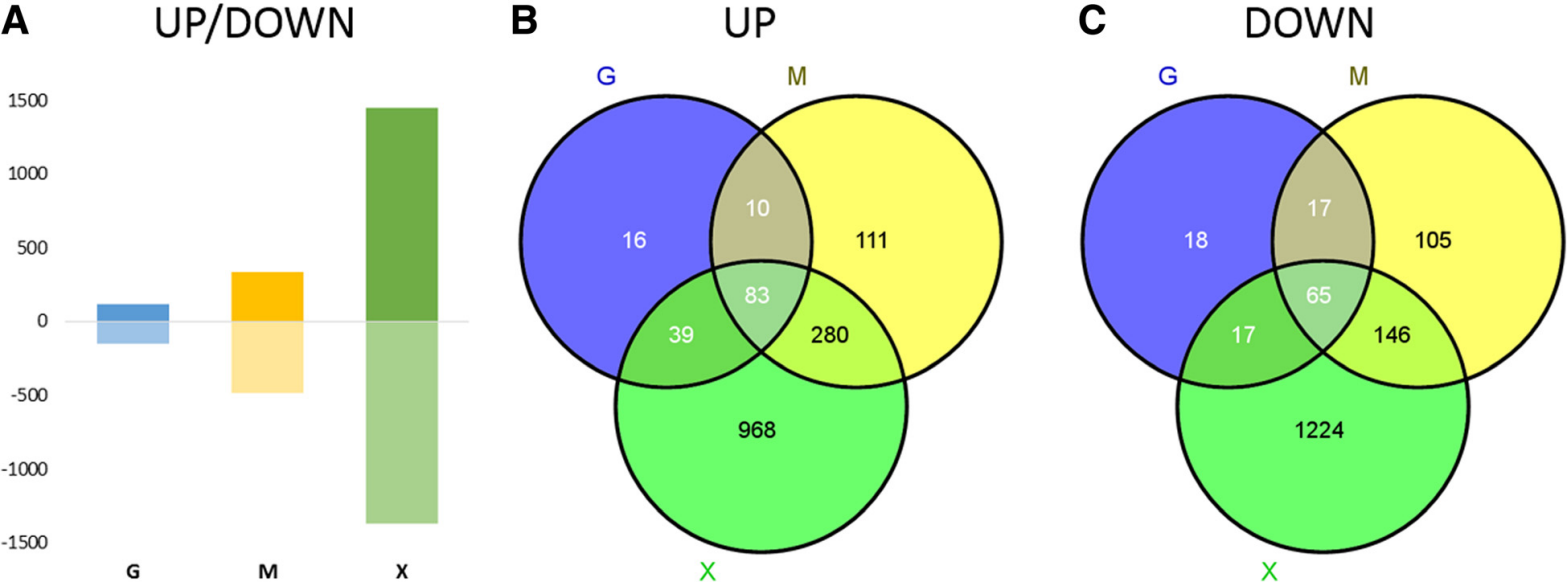
**Differentially expressed genes.** The bar chart **(A)** shows the number of differentially expressed genes in excess glycerol (G), methanol (M) and limiting glucose (X) compared to the excess glucose condition. Venn diagrams illustrate the number of up-regulated **(B)** and down-regulated genes **(C)** in the conditions and intersections. Significantly-regulated genes were identified from total RNA fold changes compared to the excess glucose condition (cutoff ±50% fold change and adjusted p-values < 0.05; [23]).

**Figure 2:**
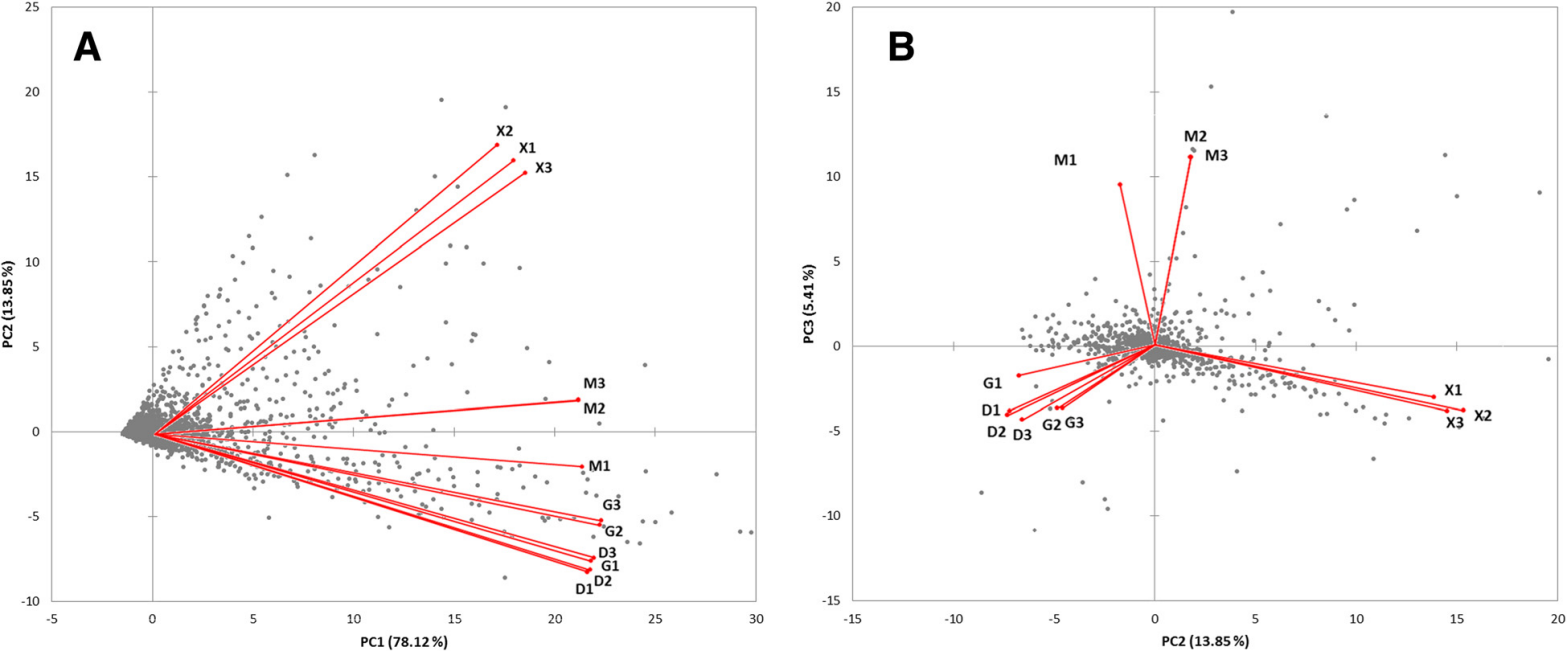
**Principal component analysis (PCA) bi-plots of microarray intensities from the green channel.** Red vectors indicate variable (condition) correlation of all analyzed replicates and the grey data points indicate observations (genes). Replicate correlation fits very well already before data normalization. The components one and two **(A)** and two and three **(B)** are compared, which explain 78, 14 and 5% of the total variation, respectively. Similar PCA biplots are obtained from microarray intensities of the red channel.

Differentially expressed genes were identified from fold changes between total RNA samples (i.e. those from unfractionated isolates). Samples from the excess glucose condition were the control for all these experiments (cut-off criteria ±50% fold change and adjusted p-values < 0.05; [23]). Transcriptional fold changes for all genes are listed in Additional file 1: Table S1. The data in Figure 1 show that cells cultured in excess glycerol (G) or glucose (D) have a very similar transcriptome with just 265 genes differentially regulated; in contrast 817 genes are differentially regulated in methanol-grown cells (M) and 2,822 are differentially regulated in glucose-limited cells (X) (Figure 1A). The corresponding Gene Ontology (GO) terms are listed in Additional file 2. A high correlation between the two excess carbon source condition transcriptomes (G and D) was also observed by principal component analysis (PCA), which showed a good correlation of the biological replicates of each condition (Figure 2). The methanol-grown and glucose-limited cells were also found to share many differentially-regulated genes and hence seem to be more similar to each other than to the two excess conditions (Figure 1B).

Further analysis (Figure 1B, C) revealed that only a small sub-set of genes are differently expressed in response to glycerol as carbon source (10% of the 148 up-regulated and 15% of the 114 down-regulated genes), while most of the regulated genes are shared either with both (56%) or at least one (approx. 30%) of the two other conditions (methanol induction or limiting glucose). We defined genes that are differentially regulated in excess glycerol conditions plus at least one other condition (either methanol induction or limiting glucose) to be subject to “glucose repression”. Genes that are differentially regulated in response to methanol induction or limiting glucose conditions, but are not differentially regulated between the two excess conditions were defined as being subject to “carbon substrate repression”.

### Polysome-mRNA association is lowest in glucose-limited cells and highest in methanol-grown cells

**Figure 3:**
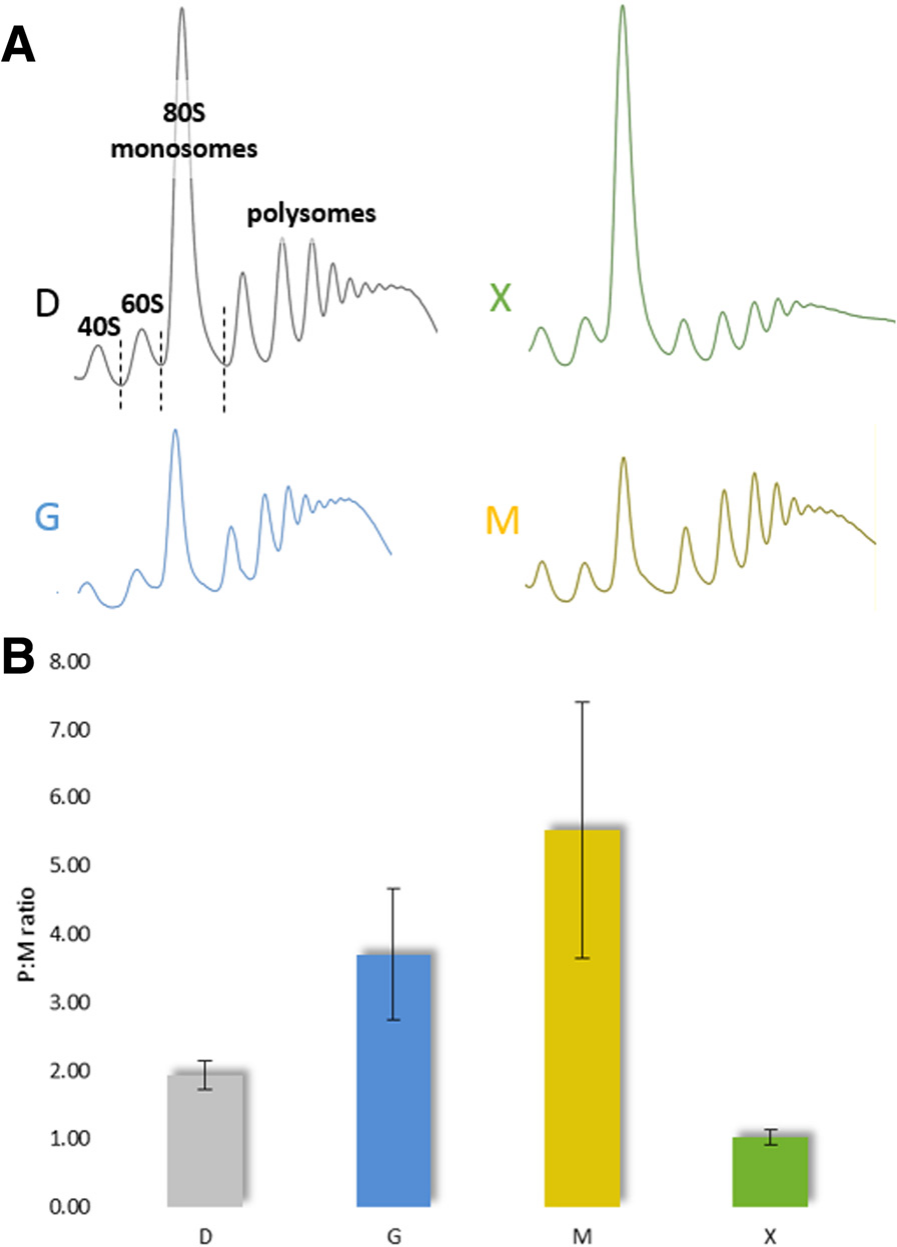
**Polysome profiles and P:M ratios for** ***P. pastoris*** **grown in different conditions. (A)** Representative polysome profiles and **(B)** a bar chart presenting P:M ratios (with sd) of the four different cultivation conditions (excess glucose, D; excess glycerol, G; limiting glucose, X; methanol, M). Corresponding peaks (40S, 60S, 80S/monosomes and polysomes) are indicated in the first (D) polysome profile. P:M ratios were calculated from areas beneath the profile curve using ImageJ.

Isolates of cells subject to the different growth conditions in Table 1 were analyzed by polysome profiling, which characterizes the translational status of a cell according to the distribution of ribosomes across the mRNA pool. Profile curves showing the proportion of ribosomes that appear as individual sub-units (40S and 60S), monosomes or polysomes (where two or more ribosomes are associated with a given mRNA transcript) are shown in Figure 3. The ratios of the polysome to monosome peak areas (P:M ratios) in the profiles (Figure 3A) are presented in Figure 3B: mRNAs that are associated with polysomes are more highly-translated than mRNAs associated with monosomes [20]. The P:M ratio is therefore established as a relative measure of translational activity at a cellular level [24,25]. In our experiments, triplicate cultures gave reproducible values for each of the different growth conditions.

Due to their similar transcript profiles, the two fastest growing conditions (excess glycerol and excess glucose, μ ~ 0.23 h^−1^) were anticipated to have similar P:M ratios. However, the excess glycerol condition had a higher P:M ratio (Figure 3) suggesting higher translational activity compared to cells grown under conditions of excess glucose. The P:M ratio was highest in cells grown on methanol, although the specific growth rate was significantly lower (μ ~ 0.10 h^−1^) compared to the excess glycerol and excess glucose conditions. The condition with the lowest specific growth rate (limiting glucose, μ ~ 0.015 h^−1^) had the lowest P:M ratio.

The transcription of translation-related genes in *P. pastoris* was previously shown to be tightly connected to growth rate in glucose-limited chemostat cultivations [3]. We found that this was also true when we analyzed the total RNA of unfractionated, slow-growing cells cultivated under limiting glucose conditions (μ ~ 0.015 h^-1^). Under these conditions, most ribosomal and translation-related genes were found to be expressed at a lower level (Additional file 1: Table S2). Strikingly, we found that those genes were equally expressed in slow-growing methanol fed cells (μ ~ 0.1 h^−1^) compared to excess glucose and glycerol (μ ~ 0.23 h^−1^), suggesting that the whole translation machinery is up-regulated despite the slow growth rate on methanol. The methanol induction-, excess glucose- and excess glycerol- conditions operated near μ_max_ for their respective condition, which means that they possess a similar μ/μ_max_ ratio. Hence, the expression of growth-associated genes might respond to the ratio of μ/μ_max_, rather than an absolute value of the specific growth rate (μ).

### Despite the general transcriptional down-regulation of translation-related genes in *P. pastoris* cells grown in limiting glucose, the transcription of certain genes is induced

Certain genes required for ribosome biogenesis and its regulation, RNA processing and translationally silent messenger ribonucleoprotein complexes (mRNPs) were highly expressed in *P. pastoris* cells grown in limiting glucose, as determined by the analysis of total mRNA (Additional file 1: Table S2): *RPS22A* (protein component of the small (40S) ribosomal subunit, homologous to mammalian ribosomal protein S15A and bacterial S8, also up-regulated in methanol-fed cells); genes linked to ribosome association, interaction or biogenesis (*TMA108, DOT6, GDE1, TMA64, PAS_FragB_0030, YMR295C, MTC1, YOR019W, MTG1*); negative regulation of RNA polymerase III transcription and TOR signaling (*KNS1*); RRPE (ribosomal RNA processing element)-binding and glucose-induced transition from quiescence to growth (*STB3*); rRNA biogenesis (*DOT6*) and mitochondrial ribosome recycling (*RRF1*). Poly(A)-binding protein is also translation-associated, and the two genes are differently expressed (PAS_chr1-4_0283 is up- and PAB1 is down-regulated) in *P. pastoris* cells grown in limiting glucose. The gene encoding the translational activator *GIS2* that was also up-regulated in limiting glucose, plays an important role as activator of mRNAs with internal ribosome entry sites [26]. It binds to a specific subset of mRNAs, associates with polysomes and localizes to RNA processing bodies (P bodies) and to stress granules. The role of cap-independent translation in physiological adaptation to stress in *S. cerevisiae* has been reported previously [27]. P bodies are used to store translationally silent mRNPs [28], and glucose-limited *P. pastoris* cells were found to differentially express related genes. *DHH1* (the gene product of which functions in de-capping and translational repression) was up-regulated, but *PAT1* and *EDC3*, with a similar function, were down-regulated in glucose-limited cells. Hence, although limiting glucose decreases global translation, certain transcripts may be translated as a part of specific stress responses.

### Growth conditions have a minimal influence on transcript-specific translational regulation

**Figure 4:**
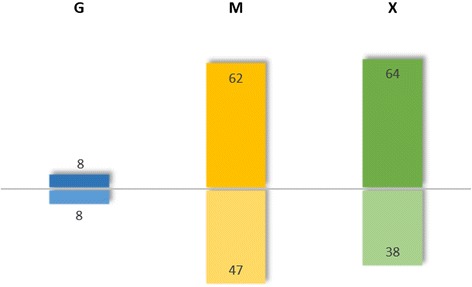
**Translationally-enriched and depleted genes.** Bar chart representing the number of translationally enriched and depleted genes in excess glycerol, limiting glucose and methanol conditions related to the excess glucose condition (cutoff ±50% change of the translational state and adjusted p-values < 0.05).

We next examined the fractionated mRNAs by microarray analysis. We normalized the abundance of each transcript in the polysome fraction to that of the total RNA, which we termed the “translational state”. In order to confirm the integrity of the RNA fractions, microarray signal intensities of the monosome, polysome and total RNA samples from the limiting glucose condition were compared as previously described [29]. The log_10_ intensity values of total RNA correlated with log_10_ of the sums of intensities in the monosome- and polysome-bound mRNA with a correlation coefficient of R^2^ = 0.963 (see Additional file 3). Translational states of individual transcripts for the excess glycerol, limiting glucose and methanol induction conditions were normalized to the excess glucose condition in order to identify transcripts with changed translational states (shown in Figure 4 and Additional file 4**)**. This identified an increased or decreased abundance of transcripts that are actively translated in the polysome fraction. Translational states of individual genes ranged from 0.08-fold (in limiting glucose conditions) to 3.05-fold (in methanol). No transcripts were totally excluded from the polysome fractions, which is in agreement with a study published by Arava et al. [30].

Only 16 transcripts had different translational states (8 increased and 8 decreased) in response to excess glycerol compared to the excess glucose condition, while more differences were found for the glucose-limited and methanol-grown cells. In excess glycerol-grown cells, *RPL2A, TEF2, RPS4B, ENO1, FBA1-1, RPL5, RPL11B* and *TDH3* had decreased translational states compared to cells grown in excess glucose. These genes are annotated with GO terms “biosynthetic/metabolic process” and “translation”. Both, the transcript level and the translational state was found to be decreased for transcripts of the glycolytic fructose 1,6-bisphosphate aldolase (*FBA1-1*), glyceraldehyde-3-phosphate dehydrogenase (*TDH3*) and phosphopyruvate hydratase (*ENO1*) in excess glycerol. This suggests that specific translational down-regulation reinforces the transcriptional down-regulation of these genes in response to excess glycerol.

In methanol-grown cells, genes required for methanol utilization (MUT), were strongly up-regulated at the transcriptional level, but had a decreased translational state compared to excess glucose. Hence translational regulation appears to counteract the strong transcriptional up-regulation of most of these genes. Such "post-transcriptional buffering" has also been observed in two *Saccharomyces* species [31]. Neither significantly enriched GO terms nor other patterns could be found in the other gene groups with altered translational states.

### Translational states are linked to ORF length and transcript abundance

We analyzed the translation states of individual transcripts compared to total mRNA for all growth conditions. Enriched gene groups were initially identified (Table 2); closer inspection revealed that the groups had closely correlated open reading frame (ORF) lengths, which has been reported previously for other organisms [32-34]. Liang et al. [2] identified *P. pastoris* gene ORFs, uORFs, UTRs and introns by sequencing, and found ORF lengths from 141 to 14853 bp, with an average of 1444 ± sd = 1032 bp (median of 1203 bp). We used this information to define three gene groups according to ORF length (Table 3): long and short genes, comprising the upper and lower quartile of all genes, and the remaining 50% of medium-length genes. Translation efficiency is also known to be affected by codon usage, so we included synonymous codon usage order (SCUO), which was obtained from the CodonO platform [35]; higher values indicate more codon bias, meaning less random codon use in a gene’s coding region. The three gene groups significantly differ in transcript level, translational states, codon usage bias (SCUO) and 5´UTR frequency: Short genes are highly transcribed (as measured by transcript abundance) and translated (high translational states), rarely possess a 5´UTR and have an enhanced codon usage bias (Table 3).

**Table 2:** **Translational regulation of functional gene groups for** ***P. pastoris*** **cells grown in excess glucose conditions**

**Functional group**	**Genes in group**	**Significantly regulated genes**	**Average translational log**_**2**_ **ratio of significantly regulated genes**	**Average ORF length of significantly regulated genes [bp]**
Secretion: chaperones	79	31	0.225	885
Antioxidant	21	7	0.160	476
Transport(er)	60	22	0.137	1669
Pexophagy	23	9	−0.082	2302
Autophagy	69	25	−0.117	1690
Vacuole	105	48	−0.151	1781
Mitochondria	110	23	−0.165	1541
TCA	20	10	−0.339	1544
Secretion: glycosylation	46	28	−0.344	1884

Average translational states and ORF length of functional gene groups for *P. pastoris* cells grown in excess glucose. Translational trends were similar in the other conditions.

**Table 3:** ***P. pastoris*** **gene statistics of long, medium and short genes**

	**Long**	**Medium**	**Short**	**All**
Number of genes	1262	2538	1265	5065
ORF length [bp]	>1807	770-1807	<770	141-14853
Mean ORF length [bp]	2786	1235	524	1444
Median ORF length [bp]	2412	1206	540	1203
Mean expression intensity	5081	7141	12092	7864
Median expression intensity	2600	2591	3416	2736
Mean SCUO	0.078	0.105	0.198	0.123
Median SCUO	0.069	0.093	0.165	0.096
Genes with 5′UTR	628	257	29	914
Genes with 5′UTR [%]	50%	10%	2%	18%
5′UTR length mean	238	253	320	245
Mean translational state	−0.22	−0.01	0.18	−0.02

Based on the information published by Liang et al. [2], all *P. pastoris* genes were split into 3 groups comprising the 25% longest (>1807 bp), the 25% shortest (<770 bp) and the remaining (50%, <1807 and >770 bp) medium length genes. Gene groups are not exactly the same size because they were split by length cut-off (some genes possess equal ORF lengths). 5′UTR information was also taken from Liang et al. [2]. Expression intensities were obtained from our total RNA microarray data which were normalized as described in the Methods section. Synonymous codon usage order (SCUO) was obtained from the CodonO platform [35].

**Figure 5:**
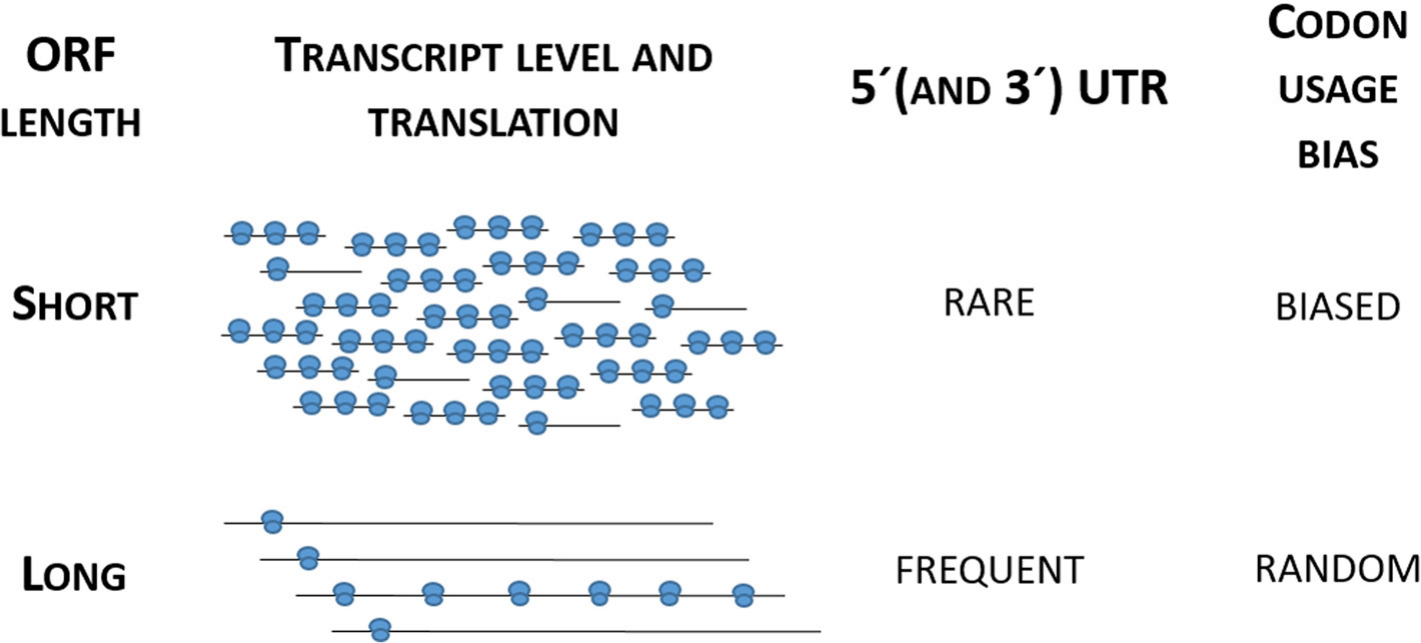
**Schematic illustration of relations between transcript level, translation, UTR frequency and codon usage bias in** ***P. pastoris*** **genes.** In contrast to genes with long coding sequences, shorter genes are more highly expressed, more efficiently translated, possess UTR’s less frequently and are more codon biased than longer genes.

Statistical tests (Fishers exact test, chi square test and regression analysis) were used to verify these relationships. ORF length was shown to have a negative correlation with transcript abundance (gene expression intensity by microarray) and codon usage bias, so short genes are more highly transcribed than longer ones (regression analysis, p-value < 1.5e^−11^) and more codon biased (non-linear regression, p-value < 2.2e^−16^). The correlation of ORF length with translational states and 5′UTR length was found to be significantly positive (p-value < 2.2e^−16^ for both). Hence, short genes are more-highly translated and rarely have a 5′UTR, while longer genes are less-highly translated and often possess a 5′UTR (Figure 5).

### Transcriptional regulation responding to different carbon sources correlates with expression of corresponding transcription factors

As mentioned above, excess glucose was used as a calibrator to calculate the transcriptional regulation in the other conditions (see Additional file 1: Table S1 for respective values for all genes). Concerning global transcriptional control systems, we could identify *P. pastoris* gene expression responding to glucose repression, carbon catabolite repression elicited by excess glucose and glycerol, as well as control by methanol availability. Limiting glucose triggers extensive transcriptional responses due to carbon limitation and low growth rate, which correlate well with the regulation patterns described by Rebnegger et al. [3] recently. Corresponding to the important role of glycogen metabolism in slow growing conditions [36], we found genes encoding glycogen synthase (*GSY2*), phosphoglucomutase (*PGM2*) and other glycogen metabolism genes (*UGP1, NTH1, ATH1, GLG1, GLC3, GLC7*) up-regulated in limiting glucose.

Glucose repression signalling is mainly mediated through the central kinase Snf1, which controls the expression of important transcription factors such as Mig1, Sip4, Rds2, Cat8 and Adr1 [37], thereby playing an important role in the utilization of non-fermentable carbon sources in *S. cerevisiae* [38]. We found the transcripts of many genes involved in catabolite (de)repression to be induced in limiting glucose, especially *CAT8-2*, which is about 39-fold up-regulated compared to excess glucose (and about 7-fold up-regulated on methanol). In addition, almost all genes that are reported to be controlled by *CAT8* in *S. cerevisiae* [39] are also up-regulated.

Interestingly, 2 homologs of Mig1 are found in the *P. pastoris* genome, one of which is about 9-fold up-regulated in response to methanol and limiting glucose (*MIG1-1*), while the second one is down-regulated on all other tested carbon sources compared to glucose (*MIG1-2*); it is possible that it acts as a carbon catabolite or glucose repressor similar to *CRE1* in *Trichoderma reesei* [40] or *CREA* in *Aspergillus nidulans* [41].

The homologue of *S. cerevisiae Activator of Ferrous Transport*, *AFT1,* was found to have induced expression levels in excess glycerol, methanol and limiting glucose conditions and has been reported to play a role in the regulation of carbon repressed genes in *P. pastoris* recently [42]. The transcription factors PAS_chr4_0324, CTH1, PAS_chr1-1_0422, PAS_chr3_1209, PAS_chr1-1_0122 were related to excess conditions*.*

Among the most strongly-induced genes in methanol and limiting glucose conditions, several transcription factors are present (Table 4). Of these, the Zn(II)2Cys6 zinc cluster protein PAS_chr3_0836, which has an 80-fold higher transcript level on methanol and 120-fold higher transcript level under limiting glucose compared to excess glucose, has significant sequence homology to *H. polymorpha MPP1* [43]. Mpp1 was suggested to be the master regulator of methanol-responsive genes in *H. polymorpha* [43,44]. Since PAS_chr3_0836 is also located in a similar chromosomal arrangement (next to *DAS1/2*; PAS_chr3_0832 and PAS_chr3_0834) to *H. polymorpha*, we propose that it is the *P. pastoris* homologue of *HpMPP1*. Pp*MXR1* encoding a transcription factor that is necessary for the activation of many genes in response to methanol [8] is induced in all three conditions compared to excess glucose. We suggest that *PpMXR1*, similar to its *S. cerevisiae* homolog *ADR1,* is needed for the activation (de-repression) of genes for alternative carbon sources including the MUT genes that are repressed in the presence of excess glucose and glycerol, but that Mpp1 is the transcriptional activator of peroxisomal import and matrix proteins required for methanol utilization in *P. pastoris*. This awaits experimental verification in future.

**Table 4:** **Transcriptional regulation of transcriptional regulators**

**Short name Pp**	**Description**	**G-D logFC**	**G-D adjPV**	**M-D logFC**	**M-D adjPV**	**X-D logFC**	**X-D adjPV**
PAS_chr4_0340	Fungal specific transcription factor domain; Zn2/Cys6 DNA-binding domain	0.35	*	**0.72**	***	**1.50**	***
*CAT8-2*	Zinc cluster transcriptional activator; necessary for derepression of a variety of genes under non-fermentative growth conditions in *S. cerevisiae*	−0.07		**2.72**	***	**5.27**	***
*YAP1*	Basic leucine zipper (bZIP) transcription factor; required for oxidative stress tolerance	0.27		**1.13**	***	**1.64**	***
PAS_chr1-4_0516	Putative transcription factor	**0.94**		**7.81**	***	**7.86**	***
*MPP1*	Fungal Zn2/Cys6 DNA-binding domain; homolog to *Hansenula polymorpha* transcription factor involved in peroxisome biogenesis/degradation	**0.90**	***	**6.34**	***	**6.99**	***
*AFT1*	Transcription factor, possibly involved in carbohydrate metabolism	**2.17**	***	**3.68**	***	**5.16**	***
*YPR022C-3*	Putative transcription factor	**1.57**	***	**2.33**	***	**4.55**	***
PAS_chr3_0348	Helix-loop-helix DNA-binding domain	0.06		0.29		**3.69**	***
*ADR1/MXR1*	Carbon source-responsive zinc-finger transcription factor, required for transcription of the glucose-repressed gene *ADH2*, of peroxisomal protein genes, and of genes required for ethanol, glycerol, and fatty acid utilization	**1.34**	***	**1.61**	***	**2.16**	***
*RSF2/ROP*	Zinc-finger protein; involved in transcriptional control of both nuclear and mitochondrial genes in *S. cerevisiae*	−0.10		**1.85**	***	−0.24	
*PpTRM1*	Zn(II)_2_Cys_6_-type transcription factor involved in the positive regulation of methanol utilization genes in P. pastoris and C. boidinii	-0.14		0.74	***	0.34	*
*SNF1*	AMP-activated serine/threonine protein kinase; found in a complex containing Snf4p and members of the Sip1p/Sip2p/Gal83p family; required for transcription of glucose-repressed genes, thermotolerance, sporulation, and peroxisome biogenesis in *S. cerevisiae*	0.39	**	**0.61**	**	**1.42**	***
*SNF2*	Catalytic subunit of the SWI/SNF chromatin remodeling complex involved in transcriptional regulation; contains DNA-stimulated ATPase activity	0.13		0.40	**	−0.37	**
*SNF4*	Activating gamma subunit of the AMP-activated Snf1p kinase complex	0.19		0.35		**0.76**	***
*MIG1-1*	Transcription factor involved in glucose repression in *S. cerevisiae*; regulated by the Snf1p kinase and the Glc7p phosphatase;	0.57	*	**1.09**	**	**3.09**	***
*MIG1-2*	Transcription factor involved in glucose repression in *S. cerevisiae*; regulated by the Snf1p kinase and the Glc7p phosphatase;	***−0.76***	**	***−1.23***	***	−0.56	***
*SIP2*	One of three beta subunits of the Snf1 kinase complex in *S. cerevisae*	0.00		−0.14		**0.65**	***
*RDS2*	Transcription factor involved in regulating gluconeogenesis and glyoxylate cycle genes; member of the zinc cluster family of proteins; confers resistance to ketoconazole in *S. cerevisiae*	−0.07		0.20		**0.83**	***
PAS_chr1-3_0274	Fungal specific transcription factor; Zn2/Cys6 DNA-binding domain	0.11		0.29		**0.90**	***
PAS_chr4_0324	Fungal specific transcription factor; Zn2/Cys6 DNA-binding domain	***−3.07***	***	***−2.99***	***	***−3.47***	***
*CTH1*	Member of the CCCH zinc finger family	***−2.54***	***	***−2.81***	***	***−2.92***	***
PAS_chr1-1_0422	Myb/SANT-like DNA-binding domain	−0.13		−0.57		***−2.56***	***
PAS_chr3_1209	Helix-loop-helix DNA-binding domain	0.16		−0.21		***−2.56***	***
PAS_chr1-1_0122	Helix-loop-helix DNA-binding domain	***−0.93***		−0.57		***−2.33***	***

Log_2_ fold changes and adjusted P-values (* adjPV < 0.1; ** adjPV < 0.05; *** adjPV < 0.01) are shown (see Additional file 1: Table S1 for detailed data). Up-regulated genes are in bold letters, down-regulated genes in bold and italics.

Other previously-characterized transcription factors acting on methanol metabolism, *ROP* (repressor of phosphoenolpyruvate carboxykinase; PAS_chr3_0554, [10]) and *TRM1* (positive regulation of methanol, PAS_chr4_0203) are induced only on methanol, but not on limiting glucose, confirming their specific involvement in methanol metabolism (reviewed by [15]).

### Glucose and carbon catabolite repression regulate the expression of genes involved in glycolysis, gluconeogenesis and the metabolism of alternative carbon sources

**Figure 6:**
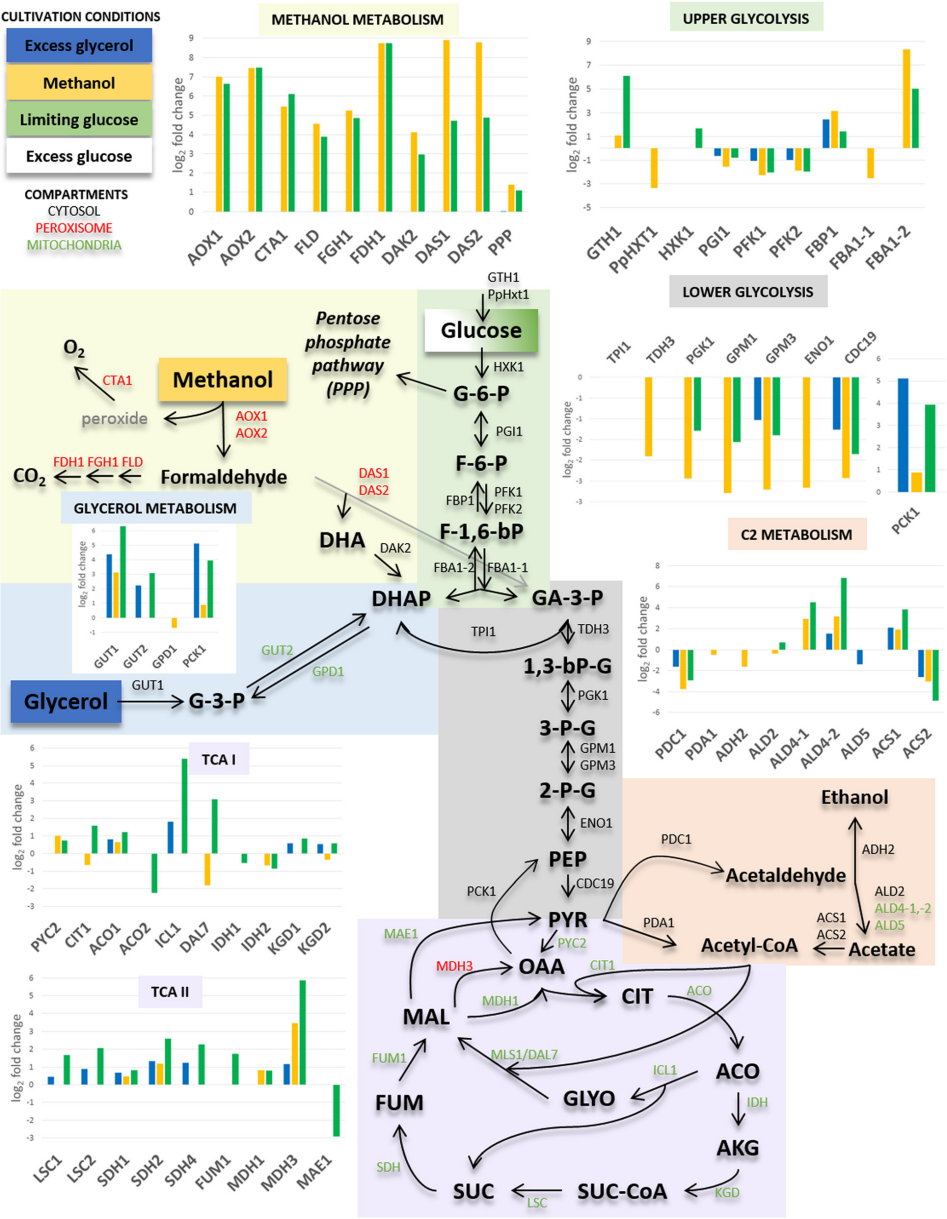
**Central carbon metabolism pathways in** ***Pichia pastoris.*** Transcriptional log_2_ fold changes of genes significantly regulated in excess glycerol, methanol and limiting glucose compared to excess glucose are presented in bar charts (cutoff ±50% fold change and adjusted p-values < 0.05; [23]). According to cellular localization, peroxisomal, cytosolic and mitochondrial enzymes are colored in red, black and green, respectively. **Metabolites:** G-6-P: glucose 6-phosphate; F-1,6-P: fructose 1,6-phosphate; DHA(P): dihydroxy acetone (phosphate); G-3-P: glycerol 3-phosphate; GA-3-P: glyceraldehyde 3-phopshate; 1,3-bPG: 1,3-bisphosphoglycerate; 3-PG: 3-phosphoglycerate; 2-PG: 2-phosphoglycerate; PEP: phosphoenolpyruvate; PYR: pyruvate; OAA: oxaloacetate; CIT: citrate; ICIT: isocitrate; AKG: alpha-keto glutarate; SUC: succinate; SUC-CoA: succinyl-Coenzyme A; FUM: fumerate; MAL: malate; GLYO: glyoxylate; **Enzymes:** AOX1/2: alcohol oxidase; CTA1: catalase A; FLD: bifunctional alcohol dehydrogenase and formaldehyde dehydrogenase; FGH1: S-formylglutathione hydrolase; FDH1: formate dehydrogenase; DAK2: dihydroxyacetone kinase; DAS1/2: dihydroxyacetone synthase; GUT1: glycerol kinase; GUT2: glycerol-3-phosphate dehydrogenase; GPD1: glycerol-3-phosphate dehydrogenase; PCK1: phosphoenolpyruvate carboxykinase; GTH1: high-affinity glucose transporter; HXT1: low-affinity glucose transporter; HXK1: hexokinase; PGI1: phosphoglucose isomerase; PFK1/2: phosphofructokinase; FBP1: fructose-1,6-bisphosphatase; FBA1-1/1-2: fructose 1,6-bisphosphate aldolase; TPI1: triose phosphate isomerase; TDH3: glyceraldehyde-3-phosphate dehydrogenase; PGK1: 3-phosphoglycerate kinase; GPM1/3: phosphoglycerate mutase; ENO1: enolase I, phosphopyruvate hydratase; CDC19: pyruvate kinase; PDC1 pyruvate decarboxylase; PDA1: E1 alpha subunit of the pyruvate dehydrogenase (PDH) complex; ALD2: cytoplasmic aldehyde dehydrogenase; ALD4-1/4-2/5: mitochondrial aldehyde dehydrogenase; ACS1/2: acetyl-coA synthetase; PYC2: pyruvate carboxylase; CIT1: citrate synthase; ACO1/2: aconitase; ICL1: isocitrate lyase; DAL7: malate synthase; IDH1/2: isocitrate dehydrogenase; KGD1: alpha-ketoglutarate dehydrogenase complex; KGD2: dihydrolipoyl transsuccinylase; LSC1: succinyl-CoA ligase; SDH1/2/4: succinate dehydrogenase; FUM1: fumarase; MDH1: mitochondrial malate dehydrogenase; MDH3: malate dehydrogenase; MAE1: mitochondrial malic enzyme.

The expression of genes related to carbon source uptake and initial metabolism is strongly regulated at the level of transcription. The respective transcriptional control of genes such as glucose sensors and transporters (low- and high-affinity), hexokinase, and glycerol- and methanol utilization are shown in Table 5 and Figure 6.

**Table 5:** **Transcriptional regulation of sugar transporters and sensors**

**Short name Pp**	**Description**	**G-D logFC**	**G-D adjPV**	**M-D logFC**	**M-D adjPV**	**X-D logFC**	**X-D adjPV**
Pp*HXT1*	*P. pastoris* major low affinity glucose transporter (major facilitator superfamily)	***−1.31***		***−3.34***	***	***−0.82***	*
*ITR2*	Myo-inositol transporter	−0.40	*	***−0.88***	*	***−0.62***	***
PAS_c034_0021	Major facilitator superfamily, related to *STL1*	***−0.59***	**	0.10		−0.55	***
PAS_chr2-1_0006	Major facilitator superfamily, Quinate permease (Quinate transporter) - similar to *S. stipitis*	−0.06		***−0.80***		−0.01	
*YBR241C*	Putative transporter, member of the sugar porter family	0.12		−0.16		0.26	
Pp*HXT2*	*P. pastoris* putative low affinity glucose transporter of the major facilitator superfamily	−0.10		−0.10		−0.09	
*STL1-1*	Glycerol proton symporter of the plasma membrane, subject to glucose-induced inactivation in *S. cerevisiae*	0.08		−0.11		**1.23**	***
*STL1-2*	Glycerol proton symporter of the plasma membrane, subject to glucose-induced inactivation in *S. cerevisiae*	−0.27		0.40		**2.08**	***
*SNF3*	*P. pastoris* plasma membrane glucose sensor Gss1, regulates glucose transport	0.16		0.44		**1.60**	***
PAS_chr3_1076	Glycerol proton symporter of the plasma membrane, related to *RGT2*	0.37		**0.65**	**	**0.62**	**
PAS_chr3_1099	Glycerol proton symporter of the plasma membrane, related to *STL1* or *RGS2*	0.34		**0.80**	**	**1.33**	***
*MAL31*	Maltose permease, high-affinity maltose transporter (alpha-glucoside transporter)	0.09		**0.81**	***	**0.68**	***
*GTH1*	*P. pastoris* major high affinity glucose transporter; similar to *K. lactis* *HGT1*	0.17		**1.09**	***	**6.14**	***
Pp*HGT1*	*P. pastoris* high affinity glucose transporter - similar to *K. lactis* *HGT1*	**0.59**		**0.86**	**	**4.91**	***
PAS_chr4_0828	Myo-inositol transporter with strong similarity to the major myo-inositol transporter Itr1p, member of the sugar transporter superfamily	**2.35**	***	**3.65**	***	**7.30**	***
*HXK1*	Hexokinase isoenzyme 1; a cytosolic protein that catalyzes phosphorylation of glucose during glucose metabolism; expression in *S. cerevisiae* is highest during growth on non-glucose carbon sources	0.30		−0.21		**1.69**	***
*HXK2*	Hexokinase isoenzyme 2; catalyzes phosphorylation of glucose in the cytosol; predominant hexokinase during growth on glucose in *S. cerevisiae*	−0.12		0.18		0.03	
*GLK1*	Glucokinase; catalyzes the phosphorylation of glucose at C6; expression regulated by non-fermentable carbon sources in *S. cerevisiae*	***−0.99***	**	***−2.58***	***	−0.34	

Log_2_ fold changes and adjusted P-values (* adjPV < 0.1; ** adjPV < 0.05; *** adjPV < 0.01) are shown (see Additional file 1: Table S1 for detailed data). Up-regulated genes are in bold letters, down-regulated genes in bold and italics.

We found glycolytic *P. pastoris* genes involved in upper and lower glycolysis to have lower expression levels in all three conditions compared to excess glucose. Glycolytic genes are known to be weakly regulated at the level of transcription in *S. cerevisiae* [45], but transcriptional regulation has been previously described for Crabtree-negative yeasts such as *P. pastoris* and *K. lactis*, and was assumed to coincide with their limited glucose uptake [46,47]. As expected, the genes encoding the key gluconeogenic enzymes fructose-1,6-bisphosphatase (*FBP1*) and PEP carboxykinase (*PCK1*) are less expressed in excess glucose (compared to the other conditions). The transition between those two pathways is associated with *Gid2/*Rmd5-dependent ubiquitin-proteasome linked elimination of the key enzyme fructose-1,6-bisphosphatase [48]. *Vid24/*GID4, encodes a previously-identified key regulator of *GID2/*Rmd5 that is strongly up-regulated in methanol fed cells. A hypothetical gene (PAS_chr1-1_0399), also strongly induced on methanol and limiting glucose, could encode the homolog of Rmd5: it contains a C3HC4 RING finger domain.

In *S. cerevisiae,* fermentative glucose- or catabolite-repressed growth is described for cells grown on excess glucose [49]. Upon glucose depletion or in the presence of non-fermentable carbon sources, such as glycerol or ethanol, extensive reprogramming of gene expression allows *S. cerevisiae* to take up alternative carbon sources and enhances activity of the glyoxylate cycle, the tricarboxylic acid (TCA) cycle and gluconeogenesis [5]. We found several *P. pastoris* genes encoding enzymes involved in the metabolism of alternative carbon sources to be less expressed during growth on glycerol, methanol and/or limiting glucose (Additional file 1: Table S3 and Figure 6). Among them, the non-annotated ORFs PAS_chr4_0338, PAS_chr4_0339 and PAS_chr4_0341 could be identified to be homologs of *LRA1*, *2* and *4*. The encoded enzymes are part of the alternative pathway of L-rhamnose catabolism present in *Pichia* (*Scheffersomyces*) *stipitis* [50] and most probably allow *P. pastoris* to utilize rhamnose as sole carbon source [51]. Interestingly, *PpLRA2* and *4* flank an uncharacterized fungal-specific Zn2/Cys6 transcription factor (PAS_chr4_0340), which is up-regulated in response to methanol and limiting glucose (Table 4). Increased transcript levels in comparison to excess glucose can also be seen for many TCA cycle genes, isocitrate lyase (*ICL1*) involved in the glyoxylate cycle (Figure 6) and genes involved in channeling alternative carbon sources into the TCA cycle (e.g. the cytosolic aldehyde dehydrogenase isoforms *ALD4-2* and PAS_chr4_0470). Interestingly, some genes encoding proteins present as isoenzymes such as *ACO1/2*, *IPD1/2* and *ACS1/2* are oppositely regulated in all the de-repressed conditions.

Respiration is repressed in excess glucose conditions during fermentative growth in *S. cerevisiae* [5,52,53], thus respiration-associated functions such as oxidative phosphorylation, mitochondrial electron transport and ATP generation are induced upon glucose depletion. Unlike *S. cerevisiae*, Crabtree-negative yeasts are dependent on respiratory processes even in excess glucose. Consequently, the expression of mitochondrial genes is not induced in the presence of non-fermentable carbon-sources in *P. pastoris* (Additional file 1: Table S4). However, several subunits of respiratory complex I [54], which is not present in *S. cerevisiae*, appear to be de-repressed.

### Methanol utilization and peroxisomal genes are subject to carbon substrate repression

Unexpectedly, the transcript levels of most genes involved in methanol utilization (MUT) are not only highly induced in methanol-grown cells but also in glucose-limited cells (Table 6). The transcript level of *AOX1* is almost equally high in both conditions. This observation correlates well with pre-induction expression from the *AOX1* promoter in the glycerol-fed batch prior to methanol addition [55-57], and high Aox1 protein levels in glucose-limited chemostats [58,59]. Repression of *AOX1* expression was previously determined in *P. pastoris* grown on glucose, glycerol, ethanol and acetate [60], with glycerol repression being specific for *P. pastoris AOX1/2*, but not for alcohol oxidase genes in related yeasts such as *H. polymorpha* or *C. boidinii* [14].

**Table 6:** **Transcriptional regulation of genes involved in methanol metabolism and peroxisome formation**

**Short name Pp**	**Description**	**G-D logFC**	**G-D adjPV**	**M-D logFC**	**M-D adjPV**	**X-D logFC**	**X-D adjPV**
*AOX1*	Alcohol oxidase (*Pichia pastoris*)	0.28		**7.00**	***	**6.64**	***
*AOX2*	Alcohol oxidase (*Pichia pastoris*)	0.39		**7.44**	***	**7.48**	***
*CTA1*	Catalase A, breaks downhydrogen peroxide in the peroxisomal matrix	**1.48**	*	**5.45**	***	**6.11**	***
*DAK2*	Dihydroxyacetone kinase, required for detoxification of dihydroxyacetone (DHA)	−0.18		**4.13**	***	**2.97**	***
*DAS1*	Dihydroxyacetone synthase variant 1	0.21		**8.91**	***	**4.72**	***
*DAS2*	Dihydroxyacetone synthase variant 2	0.10		**8.78**	***	**4.89**	***
*FDH1*	NAD(+)-dependent formate dehydrogenase, protect cells from formate	0.44		**8.74**	***	**8.75**	***
*FGH1*	S-formylglutathione hydrolase; involved in the detoxification of formaldehyde	**0.65**		**5.25**	***	**4.86**	***
*FLD*	glutathione-dependent formaldehyde dehydrogenase	0.34		**4.56**	***	**3.89**	***
*PEX1*	AAA-peroxin	0.50	***	**2.56**	***	**2.75**	***
*PEX10*	Peroxisomal membrane E3 ubiquitin ligase	0.33		**3.64**	***	**4.19**	***
*PEX11*	Peroxisomal membrane protein	**1.01**	***	**5.40**	***	**5.57**	***
*PEX12*	C3HC4-type RING-finger peroxin and E3 ubiquitin ligase	0.36	**	**2.50**	***	**3.75**	***
*PEX13*	Integral peroxisomal membrane protein	0.55	*	**4.39**	***	**3.90**	***
*PEX14*	Peroxisomal membrane peroxin	0.23		**3.14**	***	**3.90**	***
*PEX17*	Peroxisomal membrane peroxin	−0.26		**2.26**	***	**2.96**	***
*PEX19*	Chaperone and import receptor for newly-synthesized class I PMPs	−0.07		**0.75**	***	**2.10**	***
*PEX2*	RING-finger peroxin and E3 ubiquitin ligase	**0.75**	***	**3.48**	***	**3.63**	***
*PEX20*	Peroxin 20	**0.74**	***	**1.03**	***	**3.97**	***
*PEX22*	Putative peroxisomal membrane protein	0.11		0.55	*	**0.85**	***
*PEX25*	Peripheral peroxisomal membrane peroxin	−0.19		**1.09**	***	**3.29**	***
*PEX28*	Peroxisomal integral membrane peroxin	0.04		0.23		**1.55**	***
*PEX29*	Peroxisomal integral membrane peroxin	−0.24		−0.16		0.48	***
*PEX3*	Peroxisomal membrane protein (PMP)	0.37	**	**2.27**	***	**1.30**	***
*PEX30*	Peroxisomal integral membrane protein	0.10		0.09		0.47	***
*PEX31*	Peroxisomal integral membrane protein	0.36		**0.93**	*	**2.29**	***
*PEX4*	Peroxisomal ubiquitin conjugating enzyme	**0.76**	***	**2.03**	***	**4.45**	***
*PEX5*	Peroxisomal membrane signal receptor	0.29		**4.63**	***	**4.87**	***
*PEX6*	AAA-peroxin	**0.82**	***	**3.53**	***	**2.62**	***
*PEX7*	Peroxisomal signal receptor	−0.22		0.30		**1.98**	***
*PEX8*	Intraperoxisomal organizer of the peroxisomal import machinery	0.42	**	**2.93**	***	**3.52**	***
*PEX26*	Peroxisomal membrane protein	**0.94**	***	**3.16**	***	**4.63**	***
*PEX11C*	Ortholog of PEX11	0.36		**3.45**	***	**1.53**	***

Log_2_ fold changes and adjusted P-values (* adjPV < 0.1; ** adjPV < 0.05; *** adjPV < 0.01) are shown (see Additional file 1: Table S1 for detailed data). Up-regulated genes are in bold letters, down-regulated genes in bold and italics.

Although it was assumed that some MUT genes might also be regulated by catabolite de-repression [15], the extent of this regulatory pathway has not been shown experimentally in *P. pastoris.* Early observations reported that the mRNA levels of *AOX1* upon de-repression was only 1-2% of the methanol-induced mRNA levels [61], while *FLD* expression was assumed not to be under glucose repression control [62]. On the contrary we see a high level of de-repression in cells grown on limiting glucose (Table 6). This contradiction might be explained by the fact that in our set up, the cells are actively growing, while previous experiments employed glucose-exhausted stationary-phase cells for studies of de-repression. Upon (constant) methanol addition e.g. in fed batch or chemostat, MUT gene transcript levels are on average 55-fold higher compared to glucose-limited growth conditions (unpublished data). However, our data highlight that different degrees of carbon catabolite repression are acting on individual MUT genes; for example *DAS1/2* are less de-repressed than *AOX1/2*. This strongly points towards – yet unidentified –transcriptional regulators being involved in induction/repression of the individual MUT genes in addition to the global methanol regulator *PpMXR1* (summarized by [15]). Induction of peroxisomal protein synthesis was observed in *S. cerevisiae* grown on glycerol as sole carbon source [5], which appears to be different from the situation in *P. pastoris*. In the present study, up-regulation of peroxisomal gene transcript levels occurs in glucose-limited and methanol-grown cells but not in excess glycerol (Table 6), which may also be associated with the specific repression exerted by glycerol on MUT gene expression; it might be speculated that the zinc cluster protein Cat8-2 (Table 4) is the responsible transcription factor for this.

Peroxisomal processes such as methanol utilization and beta-oxidation are associated with the formation of H_2_O_2_, requiring the action of antioxidants. *YAP1*, the oxidative stress response transcription factor, and many of its target genes [63] were found to be significantly up-regulated in methanol-grown cells and/or more pronounced in limiting glucose. While it was previously shown that Yap1 is required for ROS detoxification and sufficient growth on methanol [64], the strong up-regulation of *YAP1* in glucose-limited conditions was unexpected. Interestingly, starvation is linked to the expression of genes encoding oxidative stress functions in bacteria and yeast [65,66]. The protective effect of antioxidants is proposed to have a beneficial effect in cells with nutrient limitation.

### The expression of fatty acid β-oxidation genes is up-regulated in *P. pastoris* cells responding to limiting glucose

Peroxisomal protein expression and fatty acid oxidation were previously reported to be regulated by Snf1 kinase through Adr1 action [67,68]. At least three other transcription factors act in concert with Adr1 in *S. cerevisiae* [68], but two of them – Oaf1 and Pip2 – cannot be found in *P. pastoris*. Instead, the putative fungal specific transcription factor PAS_chr1-3_0274 (Zn2/Cys6 domain) represents a homolog to FarA/B, the transcriptional activators of fatty acid utilization in *Aspergillus spp*., and *C. albicans* and *Y. lipolytica* Ctf1 [69]. The elevated transcript levels of PAS_chr1-3_0274 in limiting glucose are reflected by the strong induction of fatty acid utilization genes (e.g. all genes involved in beta-oxidation *FAA2*, *FOX2*, *POT1*, *POX1*, *ECI1*, *SPS19*, *PXA1* and *PXA2* have on average 100-fold higher transcript levels in limiting glucose, while only having approximately 2-fold higher transcript levels on methanol or glycerol in comparison to excess glucose). A similar regulation pattern was also observed for the non-annotated genes PAS_chr2-1_0249, PAS_FragB_0022, PAS_chr2-2_0403 and PAS_chr1-1_0108, indicating a possible involvement in beta-oxidation. Indeed, PAS_FragB_0022, PAS_chr2-1_0249 and PAS_chr1-1_0108 contain predicted PTS1 targeting signals [70], the latter having strong sequence homology to the peroxisome-targeted non-specific lipid transfer protein Pox18 present in *Candida tropicalis* and *Candida maltosa* [71,72]. Additionally, many genes connected to synthesis and degradation of triacylglycerol (TAG; metabolic pathway based on [73]) are regulated mainly in response to limiting glucose, which probably leads to the accumulation of free fatty acids which can then be degraded by beta-oxidation. Genes encoding fatty acid synthases (*FAS1*, *FAS2*) needed for *de novo* fatty acid biosynthesis are only up-regulated in methanol-grown cells, while all sterol biosynthesis genes with the exception of *ERG10*, which encodes the first step of the pathway (acetyl-CoA C-acetyltransferase), are down-regulated in limiting glucose. Potential interaction partners which are also strongly induced in glucose-limited and methanol-grown cells could be the putative transcription factor *SUT2* (PAS_chr1-4_0516*)* and *MPP1*, which was previously described to regulate peroxisomal matrix proteins and peroxins in *Hansenula polymorpha* [43].

## Conclusions

Our current knowledge of translational regulation comes from studies on *S. cerevisiae* cells [74-77], where stress conditions have been found to induce a global translational down-regulation that is mediated by translation initiation factors (eIFs). The specific regulation of defined mRNAs is dependent on regulatory UTR- binding protein complexes and miRNAs [78]. A significant finding emerging from this work is that the response of *P. pastoris* to different carbon sources (glycerol, glucose and methanol) is regulated mainly at the transcriptional level. Furthermore, we found translational regulation to be global rather than transcript-specific in the analyzed conditions.

Strikingly, cells grown on excess glycerol or glucose have a very similar transcriptome in contrast to the situation in *S. cerevisiae*, which undergoes extensive changes when shifting between those two catabolites [5,6]. We have also identified genes that are subject to glucose repression in *P. pastoris*. Global gene regulation patterns in glucose-limited cells differ strongly from cells grown in excess glycerol, which is a de-repressing carbon source. While this may be partly associated with the reduced growth rate of glucose-limited cells, transcriptional de-repression of genes of the methanol utilization pathway, peroxisome biogenesis and fatty acid β-oxidation is specific to glucose-limited growth (apart from methanol induction). The transcription factor(s) responsible for this regulatory function remain(s) to be identified.

Finally, we have shown that translational regulation is global rather than transcript-specific for *P. pastoris* cells in different growth conditions. Cells growing on methanol exhibited the highest P:M ratio – which might also account for the superior protein production capacities observed in this condition. Despite the lower growth rate, transcription of genes encoding ribosomal constituents and parts of the translational machinery is not affected on methanol, indicating an increased global translation which is also reflected in the degree of polysome-associated mRNAs in the polysome profiles. The high abundance of methanol utilization enzymes [14] in combination with peroxisome proliferation [79] increases the burden on the translation machinery in methanol-grown cells. Indeed, *P. pastoris* has increased cellular protein content during methylotrophic growth (Buchetics, Russmayer et al. manuscript in preparation).

## Methods

### Yeast strain and growth conditions

*Pichia pastoris* wildtype (X-33, *HIS4*^+^, Mut^+^, Invitrogen) was used for this study. In liquid culture, cells were cultivated in shake flasks at 25°C on a rotary shaker at 180 rpm. YP media without carbon source (20 g L^−1^ peptone and 10 g L^−1^ yeast extract) and synthetic media (buffered M2 minimal media, pH set to 6.0, see Delic et al. [80]) with carbon source were used for pre- and main cultures, respectively. Four different cultivation strategies (Table 1) were applied for the analysis of distinct growth phases: carbon excess (starting with 2% glycerol or glucose), methanol induction (repeated batch) or glucose-limitation (12 mm glucose feed beads, Kuhner, CH).

Cultivations with excess glycerol and glucose were inoculated to an OD of 0.1 and started with 2% carbon source, while methanol fed and glucose-limited cultivations were started with an OD of 1.5 and 0.5% or 0.25% carbon source, respectively. For the cultivation on methanol, another pulse of 0.6% methanol was given after 16 hours, about 8 hours before harvesting the culture. Limiting glucose was applied by using glucose feed beads, which are polymer particles releasing glucose at a non-linear rate of 1.63 ∙ t^0.74^ mg per disc. In order to generate a growth rate of about 0.015 h^−1^, 9 feed beads were added to 40 mL culture. The cells were harvested after 16 hours, at which time point the beads liberate 5.32 mg glucose per hour. Growth rate is calculated considering the average biomass concentration (3.3 g/L DCW), the average glucose feed rate (5.32 mg/h) and the low substrate yield coefficient Y_X/S_ (0.37 g/g) at low growth rates (see [3]). Assuming that any of the three variables would deviate up to 35%, the growth rate would still be within the range of 0.010 – 0.022 h^−1^. All cultivations were performed in triplicates and harvested at an OD of about 10 (Table 1).

### Polysome isolation and analysis

The method for polysome isolation and analysis for *P. pastoris* was adapted from previously published methods [6,19]. RNA is prone to degradation, so working with pre-cooled and RNase-free materials is required. Polysomes were fixed by the addition of 0.1 mg cycloheximide (fresh solution of 10 mg/mL DEPC water) per mL main culture (at an OD_600_ ~ 10, synthetic M2 media). The cultures were incubated for another 15 minutes on the shaker and then rapidly chilled by pouring into a 50 mL falcon tube containing 10 mL frozen DEPC-treated water and by using an ice water bath. Then the cells were recovered by 2 centrifugation steps (5300 × g, 4°C, 5 minutes) and a washing step with 10 mL cold lysis buffer (10 mM Tris–HCl pH 7.5, 0.1 M NaCl, 30 mM MgCl_2_, 50 μg/mL cycloheximide, 200 μg/mL heparin, 1% DEPC) in between. Resuspended cells (500 μL cold lysis buffer, or more if too dense) were mixed with about 1 mL baked acid washed glass beads in ribolyzer/breaking tubes and applied in a Fast Prep (pre-cooled to −80°C, Thermo Fisher Scientific, UK) for 3 minutes at 50 RPM. The lysate was transferred into fresh RNase-free tubes, cleared by centrifugation (13 K RPM, 4°C, 15 min) and analyzed using a Nanodrop spectrophotometer (Thermo Fisher Scientific, UK).

Sucrose gradients were prepared by stacking and freezing (−80°C) of each 2 mL 50%, 40%, 30%, 20% and 10% sucrose (in sucrose gradient buffer: 50 mM NH_4_Cl, 50 mM Tris-OAc pH7, 12 mM MgCl_2_) in ultracentrifuge tubes. Gradients (stored at −80°C, thawed o/n at 4°C) were carefully loaded with polysome isolate corresponding to 150 μg RNA and centrifuged at 38 K RPM and 4°C for 2 hours in a SW40 Beckman rotor. The gradient station (Biocomp, CAN) was cleaned with ethanol (70%) and DEPC-treated water prior to gradient analysis, then blanked with water and used at a speed of 0.34 mm/s. The profile was recorded and fractions were collected. ImageJ was used to calculate P:M ratios from the profiles, which is a measure of cellular translational activity.

### RNA isolation

Monosome and polysome fractions (each about 5 mL) were separated according to the live polysome profile and collected in ice-cold tubes containing 15 mL 6 M guanidine hydrochloride (resulting in ~4 M final concentration), mixed with 2.5 volumes ice-cold 100% ethanol and precipitated o/n at −20°C. Tubes were centrifuged at 3400 × g and 4°C for one hour, supernatant was removed entirely (apply short spin for residual liquid) and pellets were carefully air-dried for 5 minutes (this step can be repeated to pool material from 2 or more gradients). In order to isolate total RNA, polysome isolate corresponding to 150 μg RNA was directly mixed with guanidine hydrochloride and processed as described above. RNA was purified from the pellets using RNeasy mini kit (Qiagen, DE). Therefore, 100 μL DEPC-treated water was used for resuspension, mixed with 350 μL buffer RLT and further processed according to the manufacturer’s protocol. In the last step, 70 μL RNAse-free water was used to elute the RNA and the sample quality was checked by Nanodrop spectrophotometer and bioanalyzer analysis or gel electrophoresis.

### Microarray & data analysis

In-house *P. pastoris* DNA microarrays (Agilent platform, AMAD-ID: 034821, design and general processing as described by [23]) were used. cRNA synthesis, hybridization and scanning were done according to the Agilent protocol for 2-color expression arrays. Each sample was hybridized against an RNA reference pool sample in dye swap. The microarray data were not background normalized. Within the arrays, loess-normalization was done for the color-effect. Quantile normalization was done between the arrays, the limma package (R-project) was used to calculate fold-changes, and p-value correction was done for multiple testing using the false discovery rate controlling method of [81]. Raw microarray data are provided in Additional file 5. Venn diagrams were created using the web-based tool Venny [82] and gene ontology (GO) term enrichment analysis was conducted with GO term finder and Saccharomyces Genome Database (SGD) annotations.

Principal component analysis was performed with the Excel plug-in XLSTAT.

Synonymous codon usage order (SCUO) analysis was performed online using the CondonO platform [35].

The statistical analysis was done in R using the standard functions fisher.test, chisq.test, and lm for the regression [83]. The implementation of the Fisher test obtains the p-values directly if a 2 by 2 table is present [84], otherwise a network implementation based on FEXACT was used [85]. For the group comparisons a test on normality was performed (Shapiro-Wilk-test) and Wilcoxon-Rank tests were performed since normality was not given.

## Additional files

Additional file 1:
**Transcriptional regulation of all** *P. pastoris* **genes in excess glycerol, limiting glucose and methanol fed conditions compared to excess glucose condition is listed in Table S1.** Separate lists for genes related to translation/ribosomes/RNA processing **(Table S2),** de-repression in excess glycerol + limiting glucose and/or methanol **(Table S3),** mitochondrial genes **(Table S4).** Log_2_ fold changes and adjusted P-values (numerical values are shown in Table_S1; asterisks indicate the significance level in Table_S2-S4: * adjPV < 0.1; ** adjPV < 0.05; *** adjPV < 0.01) are shown. Additional file 2:
**Enriched GO terms in differentially expressed genes in** *P. pastoris* **cells grown in excess glycerol, limiting glucose and methanol fed cells compared to excess glucose.** Result details are provided: FDR (false discovery rate), corrected p-value and false positives. Additional file 3:
**Correlation of the log**_10_ **mean intensity of total RNA and the log**_10_ **of the sum of intensities in monosome and polysome RNA.**
Additional file 4:
**Significant translationally enriched and depleted** *P. pastoris* **transcripts in excess glycerol, limiting glucose and methanol fed cells compared to excess glucose.**
Additional file 5:
**Raw microarray data of all spot replicates on the array.** Fold changes of all sample replicates are shown from the green and red channel in relation to the reference pool sample.

## Abbreviations

G: Excess glycerol condition
D: Excess glucose condition
X: Limiting glucose condition
M: Methanol condition
μ: Specific growth rate
GO term: Gene ontology term
ORF: Open reading frame
UTR: Untranslated region
bp: Base pairs
TCA cycle: Tricarboxylic acid cycle
Pp: *Pichia pastoris*
Hp: *Hansenula polymorpha*
Sc: *Saccharomyces cervisiae*
MUT genes: Methanol utilization genes
P bodies: Processing bodies
sd: Standard deviation
adjPV: Adjusted p-value
logFC: Logarithmic (base 2) fold change
