# Supplementary figures and images for: Pichia pastoris regulates its gene-specific response to different carbon sources at the transcriptional, rather than the translational, level
Source: BMC Genomics. 2015 Mar 11;16(1):167. doi: 10.1186/s12864-015-1393-8 (PMC4408588; doi:10.1186/s12864-015-1393-8)

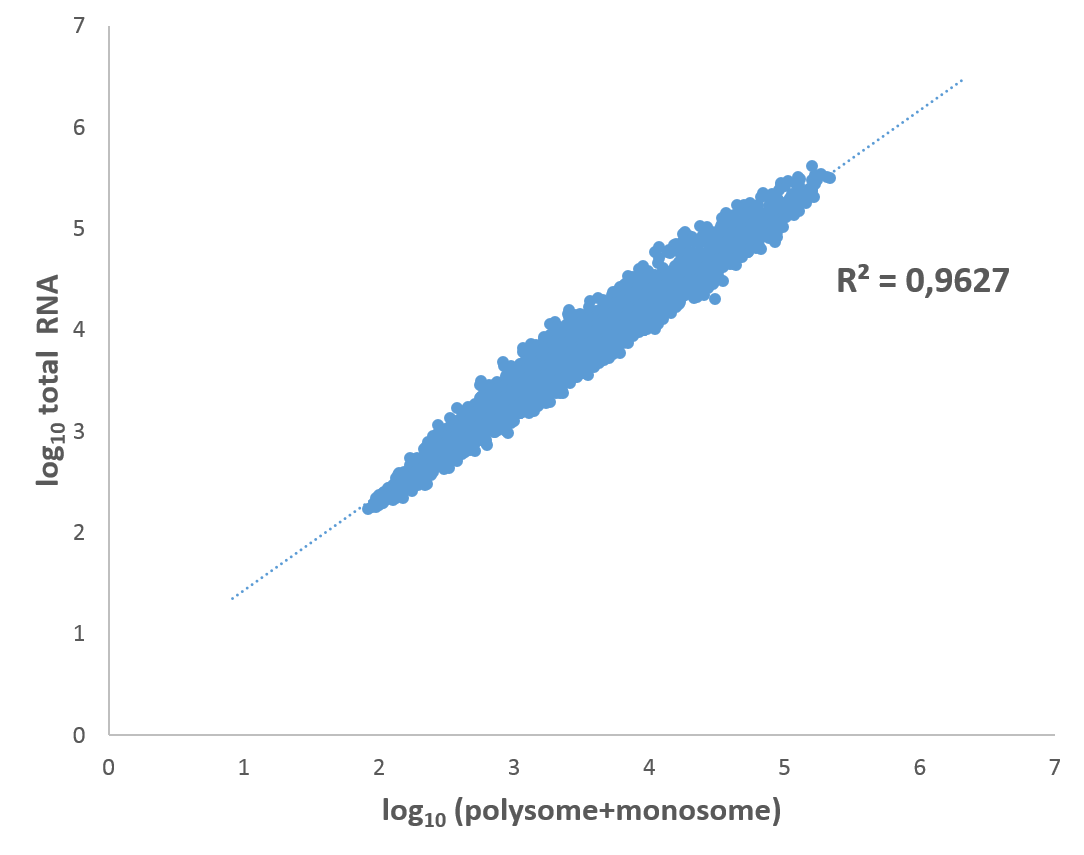

Supplement: Additional file 3: — Correlation of the log 10 mean intensity of total RNA and the log 10 of the sum of intensities in monosome and polysome RNA. [file 12864_2015_1393_MOESM3_ESM.tiff]
